# Relative Entropy in Determining Degressive Proportional Allocations

**DOI:** 10.3390/e23070903

**Published:** 2021-07-15

**Authors:** Katarzyna Cegiełka, Piotr Dniestrzański, Janusz Łyko, Arkadiusz Maciuk, Maciej Szczeciński

**Affiliations:** Wroclaw University of Economics and Business, Komandorska st. 118/120, 53-345 Wrocław, Poland; katarzyna.cegielka@ue.wroc.pl (K.C.); piotr.dniestrzanski@ue.wroc.pl (P.D.); janusz.lyko@ue.wroc.pl (J.Ł.); arkadiusz.maciuk@ue.wroc.pl (A.M.)

**Keywords:** apportionment problem, degressive proportionality, fair division, relative entropy, Theil index

## Abstract

The principle of degressively proportional apportionment of goods, being a compromise between equality and proportionality, facilitates the application of many different allocation rules. Agents with smaller entitlements are more interested in an allocation that is as close to equality as possible, while those with greater entitlements prefer an allocation as close to proportionality as possible. Using relative entropy to quantify the inequity of allocation, this paper indicates an allocation that neutralizes these two contradictory approaches by symmetrizing the inequities perceived by the smallest and largest agents participating in the apportionment. First, based on some selected properties, the set of potential allocation rules was reduced to those generated by power functions. Then, the existence of the power function whose exponent is determined so as to generate the allocation that symmetrizes the relative entropy with respect to equal and proportional allocations was shown. As a result, all agents of the apportionment are more inclined to accept the proposed allocation regardless of the size of their entitlements. The exponent found in this way shows the significant relationship between the problem under study and the well-known Theil indices of inequality. The problem may also be seen from this viewpoint.

## 1. Introduction

Allocation problems are derived from many practical issues. They concern the allocation of rights, resources, costs or burdens that are at the common disposal of participants in a given group. The object of distribution can be goods and burdens. However, the issue is often referred to as the allocation of goods, treating the release from burden as a kind of good. The goods themselves can be divisible, such as natural resources, or indivisible, such as seats in parliament or other collegiate bodies. The participants of the division, called agents, are characterized by certain attributes, called entitlements. These features determine the result of division, that is, an allocation, and are quantified but may be represented in different units depending on the nature of the problem.

Social role and the significance of allocation problems have led to many theories. These theories concern various areas of allocation problems. Examples of such areas are claims problems, cost allocation problems or apportionment problems. In a classical example of claims problems, i.e., bankruptcy issues, the value of the shared good is the bankruptcy mass expressed in currency units. Agents, also called claimants, are natural or legal persons who also have entitlements expressed in currency units, which in this case are claims such as, for example, the amount of a lost bank deposit or an unpaid liability.

Cost allocation problems concern, for example, the issue of allocating the cost of a common investment expressed in units of currency. Agents are the investors, and their entitlements can vary in size within different entities. The most common situation in practice is the presentation of entitlements in the form of opportunity costs expressed in currency units, i.e., the costs of independent action. If the result of a joint investment is to be used jointly by investors, a common definition of entitlements is the number of users on the part of each investor.

Apportionment problems originate from the division of seats in collegial bodies, for example, in the U.S. House of Representatives. In this case, agents are constituent states. The entitlement of each one is the number of its inhabitants, and the divided goods are mandates that represent legislative power.

In general, theories of allocation problems originate from common, universally accepted principles of social justice such as absolute equality or equal representation, meaning proportionality. Depending on the problem, however, various questions are formulated, and responses are given with respect to the underlying conditions that are specific to the issue. Thus, modifications of absolute equality and proportionality are allowed to take into account the underlying conditions. For example, the claims problems involve the difficulty that the resource of allocated goods does not cover the sum of the claims of the agents. Hence one assumes that the sum of claims is larger than the amount of allocated goods. This implies that the proposed implementations of the general principles underlying the distribution, as well as the rules developed on their basis, must be subject to such conditions. Therefore, the proposed rules often modify these universal principles (for example, Maimonides’ rule [1] modifies absolute equality and the contested garment rule [1] attempts to combine absolute equality with proportionality).

In problems of dividing the cost of common investments, the allocation must respect the interests of all investors and all investor coalitions. Therefore, no allocation can be proposed where one investor or their group would have to pay more than when not cooperating with others. This results again in an adjustment of the proposed allocation rules to existing assumptions and a search for equal or proportional allocations resulting from cooperation. In apportionment problems, the difficulty comes from the indivisibility of goods being allocated. As a result, the most well-known methods, such as the largest remainder method or divisor methods [2], are modifications of the principle of equal representation aiming at finding the integer allocation that respects the generally accepted rule of proportionality.

In some cases, legal regulations cause a deviation from the assumptions of absolute equality and proportionality. A well-known case is the division of seats in the European Parliament, where, if the numbers of members of the European Parliament were determined proportionally, the smallest countries of the European Union would have no representation, or the total size of the Parliament would have to be much greater than is organizationally feasible. Therefore, a degressively proportional division was decided, with deputies from more populated countries representing more citizens than those from less populated countries. This idea was regarded as so significant that it was incorporated into the Treaty of Lisbon.

Many current electoral systems can be characterized as degressively proportional either directly or indirectly. When, in political systems with bicameral legislatures, one of the chambers is elected proportionally while the other equally, then the comprehensive legislative power—being a combination of proportionality and absolute equality—can be considered degressively proportional, as in the United States Congress.

Degressive proportionality is also supported by optimization conditions. A classical case in favor of this concept is based on statistical reasoning that leads to the Penrose (square root) law, which states that the weight of a given representation should be proportional to the square root of the population represented, not to the population itself [3]. It results from the assumption that the decisions of electors in respective groups are independent random variables (see [4,5,6,7]). The reasoning presented by Theil [8] leads to similar conclusions. Based on some introduced assumptions, Theil derives a measure of frustration and shows that its minimum is attained by the allocation, which is proportional to the square root of agents’ claims. More general conclusions result from paper [9]. The utility function considered in this paper is built under the assumption that utilities of individual agents are nonnegative, increasing and concave. However, it does not suffice to uniquely determine the optimal allocation at which such a function attains its maximum. Rather, searching for the optimum is reduced to a class of degressively proportional allocations, different from proportional allocations.

Dealing with the idea of degressive proportionality in representation problems locates it near apportionment problems, whose assumptions emphasize the indivisibility of allocated goods. Even though a proportional allocation always exists, this does not mean that an integer proportional allocation exists. In a degressively proportional division of seats, we also certainly deal with the indivisibility of allocated goods, but, compared to the proportional apportionment problems, a new issue emerges, as even among non-integer divisions, there may exist more than one degressively proportional allocation. Moreover, even though there exists exactly one, mostly non-integer, the proportional allocation for every problem, still, for the same problem, there may exist infinitely many non-integer degressively proportional allocations.

This lack of uniqueness is studied in papers dealing with the so-called *unrounded degressive proportionality* (UDP), which examine the non-integer degressively proportional allocations (see for example [10,11,12,13]). In this paper, the authors mainly draw on the approach of degressively proportional allocations of divisible goods. This means that in our theoretical reflections, we do not analyze that part of the UDP problem that focuses on finding an integer representation of a given allocation, but we try to find one out of many feasible degressively proportional non-integer divisions that can be considered as satisfying a postulate of fair distribution in the best way. Integer allocation will only play its role in Section 5, devoted to the empirical validation of the proposed solution.

In Section 2, the notation used in this paper is introduced, and some basic concepts are recalled that are necessary to formulate and solve the problem, proposed in Section 3, of finding an allocation that can be regarded as fair by all agents in apportionment. In Section 4, we show that any allocation rule satisfying three properties common to equal and proportional allocation—i.e., pairwise consistency, homogeneity with respect to entitlements and homogeneity with respect to the amount of goods—can be uniquely expressed by a multiplicative function. According to a theorem proved by, among others, Theil in [14], it follows that the only nontrivial continuous multiplicative functions are power functions. Hence, the allocation problem can be reduced to an analysis of such functions.

In the area of degressively proportional allocations, we deal with one additional constraint, namely the requirement for the exponent of a power function. It turns out that it must be a number between zero and one, whereas with an exponent equaling zero, we get a rule of equal division, and with an exponent equaling one, a proportional rule. By symmetrizing the relative entropy, we get a value of the exponent that generates the allocation equally distant, in a sense, from the equal and proportional allocations, i.e., two extreme degressively proportional allocations. One can note that these two cases will be of interest to extreme groups of agents—those with the greatest entitlements will be interested in an allocation as close to proportional as possible, while agents with the smallest entitlements in an allocation as close to equal allocation as possible. By interpreting relative entropy as a measure of the inequity of two allocations, symmetrization of relative entropy leads to such an allocation that levels agents’ frustration due to throwing away the allocations they are most interested in. Therefore, the largest feasible reduction in discrimination of the smallest agents is achieved that can be accepted by the largest agents. A discussion and summary of the results are given in Section 6.

The idea to apply entropy in apportionment problems comes from Theil [14], who proposed considering it as a measure of distributional inequality. Although such an approach seems sound, in the beginning, it did not gain general acceptance. In the 21st century however, apportionment problems are again perceived through entropic eyes. Paper [15] mentions the relationship between relative entropy and well-known standard divisor methods. In paper [16], the relationship between the entire class of apportionment methods and the entire class of inequality measures was thoroughly analyzed, proving that allocations that minimize a generalized entropy are equivalent to allocations generated by divisor methods based on generalized logarithmic means.

Finally, the reader will find a deep analysis of relationships between relative entropy and apportionment problems in paper [17], where an allocation minimizing relative entropy is considered to be optimal because it is “nearest” to the proportional allocation. However, to the best of our knowledge, there are no research papers on the application of relative entropy and its symmetrization with respect to extreme feasible divisions. Hence, this is an original paper belonging to an existing strand of research.

## 2. Notation and Basic Definitions

Throughout the paper, we use the following notations. By ℕ we denote the set of all positive natural numbers, and by ${\mathbb{R}}_{+}$ we denote the set of all positive real numbers. Finite sequences are denoted with the use of parentheses for their explicit representation, e.g., $\left(p_{1},p_{2},\ldots ,p_{n}\right)$, or by bold letters, e.g., $p=\left(p_{1},p_{2},\ldots ,p_{n}\right)$. The *i*th element of the sequence p is denoted by $p_{i}$.

We consider allocation problems in which some amount of homogeneous *goods* (rights, costs, burdens, etc.) has to be divided among a finite set of n participants in the allocation, n∈ℕ. The participants of the allocation are simply called *agents*. The amount of allocated goods is denoted by h and we always assume that there is some amount of goods to be allocated, i.e., h>0. We consider problems where agents are characterized by one attribute $p_{i}$, expressed as a positive real number. This attribute is called *entitlement* and represents diverse attributes in various allocation problems, e.g., populations expressed in the number of inhabitants in the apportionment problems, or claims expressed in units of currency or savings in cost allocation problems.

We use the following notions.

An ***allocation problem*** is a pair (p,h), where p is a sequence of entitlements of agents and h is the amount of goods to be allocated. For a given (p,h) an ***allocation*** is a sequence $s=\left(s_{1},s_{2},\ldots ,s_{n}\right)$ such that $\sum_{i=1}^{n}s_{i}=h$ and for each i∈{1,2,…,n} there is $s_{i}>0$.

An ***allocation rule*** is a function $\varphi :\left(U_{n\in \mathbb{N}}{\mathbb{R}}_{+}^{n}\times {\mathbb{R}}_{+}\right)\to U_{n\in \mathbb{N}}{\mathbb{R}}_{+}^{n}$, i.e., a function assigning a unique solution $s=\varphi \left(p,h\right)\in {\mathbb{R}}_{+}^{n}$ to every allocation problem $\left(p,h\right)\in {\mathbb{R}}_{+}^{n}\times {\mathbb{R}}_{+}$. One can also grasp the allocation rule as an infinite, countable family of functions $\varphi_{i}$, i=1,2,…, such that $\varphi_{n}:{\mathbb{R}}_{+}^{n}\times {\mathbb{R}}_{+}\to {\mathbb{R}}_{+}^{n}$. Finding the allocation in this context requires two stages. First, the number of agents is determined as the length of sequence p, and then, an appropriate function $\varphi_{i}$ is selected. If rule φ assigns an allocation s to the fixed sequence p, then the amount of goods allocated to an agent with entitlements $p_{i}$, will be denoted by $\varphi \left(p,h\right)\left(p_{i}\right)$ and likewise for any subgroup of agents. For example, if φ((1,3,4),20)=(5,7,8), then φ((1,3,4),20)(3)=7 and φ((1,3,4),20)(1,4)=(5,8).

Rule φ satisfies ***pairwise consistency***, if the amount of goods allocated to any two agents with fixed entitlements depends exclusively on the sum of goods allocated to them:

(1) $$\varphi \left(p,h\right)\left(p_{i},p_{j}\right)=\left(s_{i},s_{j}\right)\Rightarrow \varphi \left(\left(p_{i},p_{j}\right),s_{i}+s_{j}\right)=\left(s_{i},s_{j}\right)$$

for all $p,s\in {\mathbb{R}}_{+}^{n}$ and $h\in {\mathbb{R}}_{+}$.

This property, in a sense, is universal because it is postulated in many allocation problems, for example, in apportionment problems, claims problems, cost allocation problems and bargaining problems in game theory. It concerns a situation where a certain division was accomplished, and some agents leave with an amount of goods allocated to them. If the allocation of the remaining goods among the remaining agents assigns to each of them the same amount of goods as in the initial division, such a rule is called *consistent*. In the paper, we consider ***pairwise consistency***, which requires this for any pair of agents. It should be emphasized that not all rules are consistent, even often used in practice. For example, in the case of apportionment problems, divisor methods are consistent but the largest remainder method, employed in the Empirical Verification section, is not consistent. The reader will find an exhaustive analysis of consistency together with its applications in [18].

Rule φ satisfies ***homogeneity with respect to entitlements***, if a proportional shift in the sequence of entitlements does not alter its value:

(2) φ(λp,h)=φ(p,h)

for all $p,s\in {\mathbb{R}}_{+}^{n}$ and $\lambda ,h\in {\mathbb{R}}_{+}$.

Rule φ satisfies ***homogeneity with respect to the amount of goods***, if a shift in the amount of goods under allocation causes a subsequent change in its value:

(3) φ(p,λh)=λφ(p,h)

for all $p,s\in {\mathbb{R}}_{+}^{n}$ and $\lambda ,h\in {\mathbb{R}}_{+}$.

The two latter properties imply that a proportional change in all entitlements should not modify the allocation, while the change in the amount of goods under allocation should adjust the allocation proportionally. In apportionment problems, the homogeneity with respect to entitlements is called briefly, *homogeneity*, and is naturally imposed by the idea of proportionality—if a proportional change of entitlements does not alter the agents’ shares in the amount of distributed goods, then it should not change the allocation [2]. Homogeneity with respect to the total amount of goods in a general case ($\lambda \in {\mathbb{R}}_{+}$) is pointless for integer allocations, but, for the same reasons as in the case of homogeneity with respect to entitlements, is also natural in apportionment problems if limited to positive integer shifts in the amount of goods.

The allocation rule that is pairwise consistent, homogeneous with respect to the amount of goods and homogenous with respect to entitlements, i.e., the rule satisfying the conditions (1)–(3), will be denoted by $\varphi^{*}$.

Rule φ satisfies the condition of ***degressive proportionality*** if

$$p_{i}\leq p_{j}\Rightarrow \left(\varphi \left(p,h\right)\left(p_{i}\right)\leq \varphi \left(p,h\right)\left(p_{j}\right)\;\wedge \;\frac{\varphi \left(p,h\right)\left(p_{i}\right)}{p_{i}}\geq \frac{\varphi \left(p,h\right)\left(p_{j}\right)}{p_{j}}\right)$$

for all i,j=1,…,n.

A degressively proportional rule is defined by two inequalities. Substituting the first one by equality, one gets the rule of equal division, whereas after replacing the second one by equality, the rule becomes the proportional rule. Thus, the degressive proportionality is situated between equality and proportionality. For this reason, symmetrization of entropy in this paper will be considered with respect to equal allocation and to proportional allocation.

Given the two allocations $s^{\prime }=\left(s_{1}^{\prime },s_{2}^{\prime },\ldots ,s_{n}^{\prime }\right)$ and $s^{\prime \prime }=\left(s_{1}^{\prime \prime },s_{2}^{\prime \prime },\ldots ,s_{n}^{\prime \prime }\right)$, ***the relative entropy from*** $s^{\prime \prime }$ **to** $\;s^{\prime }$ is defined as

(4) $$d_{KL}\left(s^{\prime },s^{\prime \prime }\right)=\sum_{i=1}^{n}\frac{s_{i}^{\prime }}{h}\log\left(\frac{s_{i}^{\prime }}{s_{i}^{\prime \prime }}\right).$$

For a given allocation problem (p,h) the allocation $s=\left(s_{1},s_{2},\ldots ,s_{n}\right)$ ***symmetrizes the relative entropy*** with respect to the allocation $s^{\prime }=\left(s_{1}^{\prime },\;s_{2}^{\prime },\ldots ,s_{n}^{\prime }\right)$ and $s^{\prime \prime }=\left(s_{1}^{\prime \prime },\;s_{2}^{\prime \prime },\ldots ,s_{n}^{\prime \prime }\right)$, if $d_{KL}\left(s^{\prime },s\right)=d_{KL}\left(s^{\prime \prime },s\right).$

The relative entropy from $s^{\prime }$ to $s^{\prime \prime }$ can be interpreted as a measure of inequity between $s^{\prime }$ and $s^{\prime \prime }$. In cases where the allocation s is sought regarding a single allocation s′, the literature recommends minimization, subject to certain conditions, of the relative entropy from s′ to s [17]. On the other hand, when the two different allocations $s^{\prime }$ and $s^{\prime \prime }$ are used as points of reference, then minimizing relative entropy regarding only one point, for example $s^{\prime }$, can result in a situation, following the use of such allocation, when the inequity perceived by agents mostly interested in the allocation $s^{\prime \prime }$ will be so great that they decline to approve it. In this context, a symmetrizing allocation can be considered the fairest one because it minimizes the inequity perceived by a group of agents mostly interested in allocation $s^{\prime }$, given the maximum level of inequity tolerated by a group of agents mostly interested in allocation $s^{\prime \prime }$ and otherwise. Moreover, the less focused the preferences of given agents are on just one allocation, the less frustration they feel after the use of an allocation that symmetrizes the relative entropy to $s^{\prime }$ and to $s^{\prime \prime }$.

## 3. Problem Statement

In allocation problems, one searches for the allocation rules that conform to generally accepted principles of fair distribution. Fairness can certainly be comprehended in various ways and is typically defined by a set of properties (sometimes called premises or axioms) that are required for the rules applied to a given allocation problem. These properties are not universal and depend on the nature of the problem (e.g., apportionment problems, claims problems, cost allocation problems). One distinguishes some desired features that have to be satisfied by the rules recognized as fair in terms of a given, actual problem, and therefore a property postulated in the apportionment problems may perhaps be pointless in the case of claims problems, etc. In claims problems, by assumption, the sum of claims exceeds the sum of goods to be distributed, and therefore it is not reasonable to propose, for example, homogeneity of the rule with respect to the amount of goods or its homogeneity with respect to entitlements, whereas these properties are required in apportionment problems. In claims problems, it is required that a simultaneous proportional shift, on a similar scale, of claims and amount of goods, should result likewise in a proportional change of the allocation sequence. It is also worth emphasizing that whether certain properties of allocation rules are considered desirable or not also depends on the individual preferences of agents or their groups. For example, it is known that postulating that several conditions are met simultaneously may narrow the circle of potential allocation rules to a proportional rule. Such a solution, compared even to an equal allocation, favors agents of greater power and is not in line with agents of lesser power. Hence, it is possible to limit the application of rules with certain properties and consider them inappropriate for solving a specific allocation problem due only to the individual preferences of specific participants of the division.

In the paper, we consider the three properties that should be satisfied. We find these properties very natural in the allocation problem under study. They are close to apportionment problems and, therefore, the required properties to be satisfied by the allocation rules, which we analyze, are derived from the solutions recognized as fair in this problem. The main principle underlying the practical solutions of apportionment problems is that of equal representation. It translates into the rule of proportional division with modifications made necessary by the condition that sequence ***s*** must be an integer. Whenever legal regulations, social solidarity or other external conditions in apportionment problems impose the replacement of a proportionality rule by another one, maintaining certain properties of a proportional division brings the proposed rule closer to standard proportionality. The choice of properties proposed in this paper is subjective. Still, it may be seen that it is justified as it leads to allocations with an attribute defined in [14] as *weak proportionality*, which means that the quotient of the amount of goods of every two agents is a fixed, not necessarily identity, function of the quotient of their entitlements, as in the case of proportionality. In addition, these properties are characteristic not only of a proportional allocation but also an equal allocation, which represents the other extreme case of degressive proportionality.

The problem addressed in this paper is to find a degressively proportional allocation that can be recognized as fair, or at least as non-harming to agents, taking into account the amount of goods allocated to other agents. This problem can be reduced to finding the exponent β∈[0,1] of a power function that determines the allocation. The analysis of power functions in this context is not a new approach. For example, in papers [3,19,20], the exponent β=0.5 is proposed for the use in allocation. Interestingly enough, the arguments in favor of the fairness of such a solution are not the same. Penrose [3] employs an assumption of voters’ independent decisions, Theil and Schrage [19] minimize the average number of citizens represented by the Parliament members from each country, whereas Dniestrzański [20] examines the conditions to be satisfied by the derivative of the allocation function in degressively proportional division.

As can be seen, the fairness of a solution is demonstrated using diverse properties from different theoretical concepts and methods from various areas of mathematics. These proposals typically locate fair division in the middle of the interval [0,1], i.e., they find the solution precisely in the middle of the examined interval that symmetrizes, in a sense, the set of feasible solutions. The idea of how to find the fair division proposed in this paper is similar. As a criterion for selecting the exponent β we assume the postulate of symmetrization of the relative entropy, also called the Kullback–Leibler divergence measure with respect to the distance between equal and proportional allocations. The symmetrization refers to the above-mentioned proposals, with the emphasis put on the equality of the relative entropies with respect to the extreme allocations, equal and proportional, achieved for β=0 and β=1, respectively—instead of the absolute center.

## 4. Results

The assumptions of pairwise consistency, homogeneity with respect to entitlements and homogeneity with respect to the amount of goods significantly reduce the range of potential allocation rules. We shall prove that each allocation rule $\varphi^{*}$ generates a multiplicative function $g:{\mathbb{R}}_{+}\to {\mathbb{R}}_{+}$, defined on quotients of agents’ entitlements, where a multiplicative function means any function satisfying the relationship

(5) g(xy)=g(x)g(y).

Moreover, a reverse implication also holds, namely, each multiplicative function determines precisely one allocation rule $\varphi^{*}$ in such a way that if its argument is a quotient of entitlements of any two agents, then its value equals the quotient of goods allocated to them by this rule. As a result, the allocation problem satisfying the three mentioned properties of the fair distribution, which are derived from the proportionality rule, can be reduced to an analysis of multiplicative functions.

**Proposition** **1.** *For each rule*$\varphi^{*}$there exists a multiplicative function$f_{\varphi^{*}}:{\mathbb{R}}_{+}\to {\mathbb{R}}_{+}$*such that if*$\varphi^{*}\left(p,h\right)=s$*then*$\frac{s_{i}}{s_{j}}=f_{\varphi^{*}}\left(\frac{p_{i}}{p_{j}}\right)$*for any indices*i,j.

**Proof.**  For given p,h let $\varphi^{*}\left(p,h\right)=s$ and $\varphi^{*}\left(p,h\right)\left(p_{i},p_{j}\right)=\left(s_{i},s_{j}\right)$. Then

$$\begin{array}{ll} \frac{s_{i}}{s_{j}}=\frac{\varphi^{*}\left(p,h\right)\left(p_{i}\right)}{\varphi^{*}\left(p,h\right)\left(p_{j}\right)} & =\frac{\varphi^{*}(\left(p_{i},p_{j}),s_{i}+s_{j}\right)\left(p_{i}\right)}{\varphi^{*}(\left(p_{i},p_{j}),s_{i}+s_{j}\right)\left(p_{j}\right)}=\frac{(s_{i}+s_{j})\varphi^{*}(\left(p_{i},p_{j}),1\right)\left(p_{i}\right)}{(s_{i}+s_{j})\varphi^{*}(\left(p_{i},p_{j}),1\right)\left(p_{j}\right)}\\ & =\frac{\varphi^{*}(\left(1,\frac{p_{i}}{p_{j}}),1\right)\left(1\right)}{\varphi^{*}(\left(1,\frac{p_{i}}{p_{j}}),1\right)\left(\frac{p_{i}}{p_{j}}\right)}=f_{\varphi^{*}}\left(\frac{p_{i}}{p_{j}}\right). \end{array}$$

The second equality results from pairwise consistency, the third one from homogeneity with respect to the amount of goods, the fourth one from homogeneity with respect to the entitlements.

Since $f_{\varphi^{*}}\left(\frac{p_{i}}{p_{j}}\right)=\frac{\varphi^{*}\left(\left(1,p_{i}/p_{j}\right),1\right)\left(1\right)}{\varphi^{*}\left(\left(1,p_{i}/p_{j}\right),1\right)\left(p_{i}/p_{j}\right)}$, $f_{\varphi^{*}}$ is a uniquely determined function. Additionally, if $\varphi^{*}$ assigns the amount of goods $s_{i},s_{k},s_{j}$ to agents with entitlements $p_{i},p_{k},p_{j}$, respectively, then for $x=\frac{p_{i}}{p_{k}}$ and $y=\frac{p_{k}}{p_{j}}$ we have

$$f_{\varphi^{*}}\left(xy\right)=f_{\varphi^{*}}\left(\frac{p_{i}}{p_{k}}\frac{p_{k}}{p_{j}}\right)=f_{\varphi^{*}}\left(\frac{p_{i}}{p_{j}}\right)=\frac{s_{i}}{s_{j}}=\frac{s_{i}}{s_{k}}\frac{s_{k}}{s_{j}}=f_{\varphi^{*}}\left(\frac{p_{i}}{p_{k}}\right)f_{\varphi^{*}}\left(\frac{p_{k}}{p_{j}}\right)=f_{\varphi^{*}}\left(x\right)f_{\varphi^{*}}\left(y\right),$$

and hence, $f_{\varphi^{*}}$ is multiplicative. □

Note that $f_{\varphi^{*}}\left(1\right)=1$ holds for each allocation rule $\varphi^{*}$, as well as the continuity of $f_{\varphi^{*}}$ results from continuity of $\varphi^{*}$.

**Proposition** **2.** *Let* $g:{\mathbb{R}}_{+}\to {\mathbb{R}}_{+}$ *be a multiplicative function. Let* φ *be a rule that assigns an allocation* $s=\left(s_{1},s_{2},\ldots ,s_{n}\right)$ *to each allocation problem* (p,h) *, so that* $\frac{s_{i}}{s_{j}}=g\left(\frac{p_{i}}{p_{j}}\right)$ *holds for any* i,j∈{1,2,…,n} *. Then the rule* φ *satisfies the conditions (1)–(3) and is of the form*

(6) $$\varphi \left(p,h\right)=s=\frac{h}{\sum_{i=1}^{n}g\left(p_{i}\right)}\left(g\left(p_{1}\right),g\left(p_{2}\right),\ldots ,g\left(p_{n}\right)\right).$$

**Proof.**  Since $\frac{s_{i}}{s_{j}}=g\left(\frac{p_{i}}{p_{j}}\right)$ and g is a multiplicative function, we have $s_{i}=g\left(\frac{p_{i}}{p_{1}}\right)s_{1}=\frac{g(p_{i})}{g\left(p_{1}\right)}s_{1}$. Hence $s=\left(s_{1},s_{2},\ldots ,s_{n}\right)=\frac{s_{1}}{g\left(p_{1}\right)}\left(g\left(p_{1}\right),g\left(p_{2}\right),\ldots ,g\left(p_{n}\right)\right)$. It suffices to take $s_{1}=\frac{hg\left(p_{1}\right)}{\sum_{i=1}^{n}g\left(p_{i}\right)}$ so as to get the sum of the elements of this sequence equal to h and hence the rule is well defined. Thus, the rule is of the form

$$\varphi \left(p,h\right)=s=\frac{h}{\sum_{i=1}^{n}g\left(p_{i}\right)}\left(g\left(p_{1}\right),g\left(p_{2}\right),\ldots ,g\left(p_{n}\right)\right).$$

The quotient $\frac{s_{i}}{s_{j}}$ is a function of the quotient $\frac{p_{i}}{p_{j}}$, hence it is constant for a given p, which implies pairwise consistency. Homogeneity with respect to the amount of goods follows from the relationship

$$\varphi \left(p,\lambda h\right)\left(p_{i}\right)=\frac{\lambda h}{\sum_{i=1}^{n}g\left(p_{i}\right)}g\left(p_{i}\right)=\lambda \frac{h}{\sum_{i=1}^{n}g\left(p_{i}\right)}g\left(p_{i}\right)=\lambda \varphi \left(p,h\right)\left(p_{i}\right).$$

Since the quotients of appropriate elements of the sequences p and λp are equal, we have φ(λp,h)=φ(p,h), which proves the homogeneity with respect to entitlements. □

Let us denote by $\varphi_{g}^{*}$ the rule defined by formula (6). If g(x)≡1, then $\varphi_{g}^{*}$ is the rule of equal allocation, and when g(x)=x the rule is proportional. From Proposition 1 and Proposition 2 it follows that one can consider the rules $\varphi^{*}$ in two ways. On the one hand each rule $\varphi^{*}$ generates a certain multiplicative function $f_{\varphi^{*}}$, while on the other hand, each multiplicative function $g:{\mathbb{R}}_{+}\to {\mathbb{R}}_{+}$ uniquely generates a certain rule $\varphi_{g}^{*}$. This function will be called a **generating function** of allocation $\varphi_{g}^{*}$. Rule $\varphi_{g}^{*}$ preserves the ordering of entitlements, i.e., a greater agent is allocated more goods than a smaller agent if, and only if, g is an increasing function, otherwise, the rule $\varphi_{g}^{*}$ for a decreasing function reverses the ordering of entitlements. As will be seen subsequently, if function g is continuous, it is either monotonic or constant, therefore rule $\varphi_{g}^{*}$ either preserves the ordering of entitlements or reverses it, or it is the rule of equal allocation. Certainly, the continuity of function g implies continuity of rule $\varphi_{g}^{*}$ generated by this function.

The continuity of allocation rules is emphasized in many papers. In case of a claims problem or division of burdens, e.g., taxes, continuity is especially highlighted. The rules applied to these issues should not, for obvious reasons, lead to incremental changes of burdens [1,21]. In practice, minor changes in the size of entitlements of individual agents involve minor changes in the amount of goods received by them or in imposed burdens. This, in turn, means that small changes in the quotients of entitlements will cause small changes in the quotients of the amount of distributed goods and chores.

After analyzing allocations as being a function of the quotients of entitlements, Theil defined a so-called weak proportionality condition (see [14]). In this paper, Theil also proved the proposition that follows.

**Proposition** **3** **(Theil** **1969).** *The only continuous solutions to a functional equation*g(xy)=g(x)g(y)*are functions of the form*$\left(x\right)=x^{\beta }$*, where*β∈ℝ*or*g≡0.

It follows from Proposition 3 that the only generating functions for the pairwise consistent rules—homogeneous with respect to the amount of goods and homogenous with respect to entitlements—are power functions. We get a more restrictive constraint in degressively proportional rules. This constraint is shown in Corollary 1.

**Corollary** **1.** *The rule*$\varphi_{g}^{*}$generated by the function$g\left(x\right)=x^{\beta }$*satisfies the condition of degressive proportionality if and only if*0≤β≤1.

**Proof.**  It follows from Proposition 2 that $\varphi \left(p,h\right)\left(p_{i}\right)=s_{i}=\frac{p_{i}^{\beta }}{p_{1}^{\beta }+p_{2}^{\beta }+\ldots +p_{n}^{\beta }}$ holds for any i∈{1,2,…,n}. Set i,j∈{1,2,…,n} so that $p_{i}\leq p_{j}$. The condition of degressive proportionality means that $s_{i}\leq s_{j}$ and $\frac{s_{i}}{p_{i}}\geq \frac{s_{j}}{p_{j}}$. We have

$$s_{i}\leq s_{j}\Leftrightarrow \frac{p_{i}^{\beta }}{p_{1}^{\beta }+p_{2}^{\beta }+\ldots +p_{n}^{\beta }}\leq \frac{p_{j}^{\beta }}{p_{1}^{\beta }+p_{2}^{\beta }+\ldots +p_{n}^{\beta }}\Leftrightarrow p_{i}^{\beta }\leq p_{j}^{\beta }\Leftrightarrow \beta \geq 0$$

and

$$\frac{s_{i}}{p_{i}}\geq \frac{s_{j}}{p_{j}}\Leftrightarrow \frac{s_{i}}{s_{j}}\geq \frac{p_{i}}{p_{j}}\Leftrightarrow \frac{p_{i}^{\beta }}{p_{j}^{\beta }}\geq \frac{p_{i}}{p_{j}}\Leftrightarrow {\left(\frac{p_{i}}{p_{j}}\right)}^{\beta }\geq \frac{p_{i}}{p_{j}}\Leftrightarrow \beta \leq 1\;.\;□$$

Let $g\left(x\right)=x^{\beta }$ be a generating function of rule $\varphi_{g}^{*}$. Notice that for β=0 rule $\varphi_{g}^{*}$ yields an equal allocation and for β=1 a proportional allocation. Hence the conclusion that each degressively proportional rule generated by the function *g* is, in a sense, an intermediate solution between equality and proportionality. The exponent β shows whether it is closer to equality or to proportionality. With no additional indications concerning the practical use of a given exponent, one may search for compromise solutions that symmetrize the problem under study. Justifying such proposals based on fair division is usually allowed. One of the possible proposals in this spirit is the symmetrization of relative entropy.

**Proposition** **4.** *For any allocation problem*(p,h)*there exists exactly one value of parameter*β*, such that the allocation*$s=\frac{h}{\sum_{i=1}^{n}p_{i}^{\beta }}\left(p_{1}^{\beta },\;p_{2}^{\beta },\ldots ,p_{n}^{\beta }\right)$*symmetrizes a relative entropy with respect to equal and proportionality allocations. Moreover,*

(7) $$\beta =\frac{T_{T}\left(p\right)}{T_{T}\left(p\right)+T_{L}\left(p\right)},$$

*where*$\bar{p}=\frac{1}{n}\sum_{i=1}^{n}p_{i}$, $T_{T}\left(p\right)=\frac{1}{n}\sum_{i=1}^{n}\frac{p_{i}}{\bar{p}}\log\left(\frac{p_{i}}{\bar{p}}\right)$*is the Theil entropy index and*$T_{L}\left(p\right)=-\frac{1}{n}\sum_{i=1}^{n}\log\left(\frac{p_{i}}{\bar{p}}\right)$*is the mean log deviation index (MLD).*

**Proof.**  One can note that $s=\frac{h}{c}\left(p_{1}^{\beta },\;p_{2}^{\beta },\ldots ,p_{n}^{\beta }\right)$, where $c=\sum_{i=1}^{n}p_{i}^{\beta }$. Let $s_{equal}=\frac{h}{n}\left(1,1,\ldots ,1\right)$ and $s_{prop}=\frac{h}{\sum_{i=1}^{n}p_{i}}p=\frac{h}{n\bar{p}}p$ be equal and proportional allocations for the allocation problem (p,h). Formula (4) yields the following:

$$\begin{array}{c} d_{KL}\left(\;s_{equal},\;s\right)=\sum_{i=1}^{n}\frac{1}{n}\log\left(\frac{h}{nc}p_{i}^{-\beta }\right)=\log\left(h\right)-\log\left(n\right)-\log\left(c\right)-\beta \frac{1}{n}\sum_{i=1}^{n}\log\left(p_{i}\right),\\ \;\;\;d_{KL}\left(\;s_{prop},\;s\right)=\sum_{i=1}^{n}\frac{p_{i}}{n\bar{p}}\log\left(\frac{hp_{i}}{n\bar{p}c}p_{i}^{-\beta }\right)\\ \;\;\;=\log\left(h\right)-\log\left(n\right)-\log\left(c\right)+\frac{1}{n}\sum_{i=1}^{n}\frac{p_{i}}{\bar{p}}\log\left(\frac{p_{i}}{\bar{p}}\right)-\beta \frac{1}{n}\sum_{i=1}^{n}\frac{p_{i}}{\bar{p}}\log\left(p_{i}\right). \end{array}$$

It is easily seen that equation $d_{KL}\left(s_{equal},\;s\right)=d_{KL}\left(s_{prop},\;s\right)$ with respect to β has exactly one solution:

$$\begin{array}{c} \beta =\frac{\frac{1}{n}\sum_{i=1}^{n}\frac{p_{i}}{\bar{p}}\log\left(\frac{p_{i}}{\bar{p}}\right)}{\frac{1}{n}\sum_{i=1}^{n}\frac{p_{i}}{\bar{p}}\log\left(p_{i}\right)-\frac{1}{n}\sum_{i=1}^{n}\log\left(p_{i}\right)}\\ \;\;\;\;\;\;\;=\frac{\frac{1}{n}\sum_{i=1}^{n}\frac{p_{i}}{\bar{p}}\log\left(\frac{p_{i}}{\bar{p}}\right)}{\frac{1}{n}\sum_{i=1}^{n}\frac{p_{i}}{\bar{p}}\left(\log\left(\frac{p_{i}}{\bar{p}}\right)+\log\left(\bar{p}\right)\right)-\frac{1}{n}\sum_{i=1}^{n}\left(\log\left(\frac{p_{i}}{\bar{p}}\right)+\log\left(\bar{p}\right)\right)}\\ \;\;\;\;\;\;\;=\frac{\frac{1}{n}\sum_{i=1}^{n}\frac{p_{i}}{\bar{p}}\log\left(\frac{p_{i}}{\bar{p}}\right)}{\frac{1}{n}\sum_{i=1}^{n}\frac{p_{i}}{\bar{p}}\log\left(\frac{p_{i}}{\bar{p}}\right)+\log\left(\bar{p}\right)-\frac{1}{n}\sum_{i=1}^{n}\log\left(\frac{p_{i}}{\bar{p}}\right)-\log\left(\bar{p}\right)}\\ \;\;\;\;\;\;\;=\frac{\frac{1}{n}\sum_{i=1}^{n}\frac{p_{i}}{\bar{p}}\log\left(\frac{p_{i}}{\bar{p}}\right)}{\frac{1}{n}\sum_{i=1}^{n}\frac{p_{i}}{\bar{p}}\log\left(\frac{p_{i}}{\bar{p}}\right)-\frac{1}{n}\sum_{i=1}^{n}\log\left(\frac{p_{i}}{\bar{p}}\right)}=\frac{T_{T}\left(p\right)}{T_{T}\left(p\right)+T_{L}\left(p\right)}.□ \end{array}$$

Since $0\leq \frac{T_{T}\left(p\right)}{T_{T}\left(p\right)+T_{L}\left(p\right)}\leq 1$, the allocation determined in this way is degressively proportional.

It should be mentioned that relative entropy is not symmetric, i.e., $d_{KL}\left(s^{\prime }\;,s^{\prime \prime }\right)\neq d_{KL}\left(s^{\prime \prime }\;,s^{\prime }\right)$. An allocation symmetrizing relative entropy with respect to equal allocation and to proportional allocation could also be considered as an allocation-satisfying condition $d_{KL}\left(s,s_{equal}\right)=d_{KL}\left(s,s_{prop}\right)$, but the solution of this equation can only be obtained with the use of sophisticated numerical methods.

## 5. Empirical Verification

The allocation symmetrizing relative entropy with respect to equal and proportional allocation was applied to distribute the seats in the European Parliament of the ninth term, 2019–2024. Following the withdrawal of the United Kingdom from the European Union in this period, the computation was performed for the state after Brexit. This means that the total number of seats to be distributed among 27 countries was 705. Sequence p (Table 1, column 2) describing the populations of member states was taken from [22].

For this sequence of populations, exponent β was found to be approximately 0.4476, and the allocation is of the form $s=\left(s_{1},\ldots ,s_{27}\right)$, where $s_{i}=\frac{705}{\sum_{i=1}^{27}{\left(\frac{p_{i}}{434403}\right)}^{0.4476}}\cdot {\left(\frac{p_{i}}{434403}\right)}^{0.4476}$ (see Table 1, column 4). The problem of seat division in the European Parliament is an integer division problem, and hence this allocation is merely a starting point to indicate its integer representation. Table 1 presents a proposed integer allocation (s) that minimizes relative entropy with respect to allocation s, i.e., from all integer allocations whose sum is 705, allocation (s), such that its relative entropy with respect to s is minimal, has been chosen. In this case, it turns out that it is identical to the allocation obtained from the largest remainder method.

It has to be noted that this allocation satisfies the boundary conditions imposed by the Treaty of Lisbon, i.e., the number of seats allocated to the smallest country (Malta) is not less than six and the number of seats allocated to the largest country (Germany) does not exceed 96. Hence this solution can be accepted from the legal viewpoint. In addition, it properly renders the idea of European solidarity because it represents the interests of less populated EU member states to a greater degree than the current allocation. Table 1 shows that medium-populated countries would mostly gain (from four to six seats, with the largest gain realized in the case of Belgium—six seats), if the current division of seats were replaced by the solution determined from the allocation symmetrizing the relative entropy with respect to equal and proportional allocations. The number of allocated seats would decrease only in the case of the five largest countries, with the greatest losses in the number of seats allocated to France, Italy and Germany. The only country that would not feel any change is Malta.

Note that the final integer allocation depends on the choice of the rounding method. Moreover, fulfillment of the condition of degressive proportionality is guaranteed at the level of non-integer division, the rounding of which may violate this condition. This is the case in the given example for the country pairs Finland–Denmark and Portugal–The Czech Republic. In the context of the European Parliament, however, this is without prejudice to the findings in the European Parliament Resolution. According to them, the condition of degressive proportionality is to be met before rounding to integers, i.e., in the analyzed case for sequence s.

## 6. Discussion and Conclusions

Many allocation rules in the literature and in practice are based on two core notions of justice: equality and proportionality. When the agents’ entitlements are undisputed, an equal or proportional allocation is the simplest and most applied solution to the problem, depending on the nature of the problem and possible constraints resulting, e.g., from the legal environment or prior arrangements accepted by agents. Another significant group of rules embraces all sorts of intermediary solutions that are a compromise between equality and proportionality, e.g., Maimonides’ rule, Aumann–Maschler rule [1], or the adjusted proportional rule [1]. In this area, we deal with, for example, methods where a part of goods are equally distributed among agents, with another part being allocated proportionally. Yet another approach is based on the assumption that not only the benefits are to be distributed, but also the losses, which are defined as the differences between entitlements and actual allotments assigned to the agents as a result of division.

Degressive proportionality likewise walks a line between equality and proportionality. Its basic advantage consists in accurately defining what this “in-between” status means: that the quotient of the amount of allocated goods and entitlements is nonincreasing with respect to the increasing sequence of agents’ entitlements. On the other hand, accurately positioning the degressive proportionality between equality and proportionality does not provide a unique solution to the problem of goods apportionment. In many instances, there may exist a lot of degressively proportional allocations, even, in the case of divisible goods, infinitely many.

It has been proven in the paper that the three basic properties of equal and proportional allocation rules (pairwise consistency, homogeneity with respect to entitlements and homogeneity with respect to the amount of goods) determine a coherent class of allocation rules generated by the power functions, i.e., the unique continuous and multiplicative generating functions. Constraining the exponent of the power function to the unit interval (i.e., the interval whose endpoints determine the equal allocation rule and proportional allocation rule), rules satisfying the condition of degressive proportionality were obtained. In such a constrained class, an allocation that reconciles the contradictory interests of agents with discrepant entitlements participating in distribution was indicated. This was done by leveling the inequity that can be a result of the discrepancies and disparities in the amount of allocated goods. In addition, it has been proven in this paper that the exponent of the relevant power function, in this case, is closely linked with two Theil indices. The results obtained have the prospect to be applied in many problems involving the distribution of divisible and indivisible goods. In the case of indivisible goods, such as the distribution of seats in collegial bodies, the method of determining an integer representation of the indicated allocation remains an open question. The solution proposed in this paper consists of indicating an allocation that minimizes relative entropy, and it is identical with the solution obtained by means of the largest remainder method, i.e., the minimization of the Euclidean distance. That is not always the case, however, and the examples given are not the only ways to solve this problem, thus implying a possible extension of the research into integer allocations. More research could also embrace a deeper analysis of the relationships between the results obtained here and the Theil or other indices of inequality.

Another interesting topic for further research is an analysis of symmetrization of the complementary dual function of relative entropy, that is, relative extropy introduced in [23]. Since entropy and extropy are dual, including extropy in considerations about the inequity of allocation seems to be appropriate and promising in the context of potential results, similarly for other types of relative entropy based on generalized entropies [24].

## Figures and Tables

**Table 1:** Non-integer allocation of seats in the European Parliament determined by the allocation s and its integer representation (s).

Country	Population	Current	*s*	(*s*)
Malta	434,403	6	6.18332642	6
Luxemburg	576,249	6	7.0169732	7
Cyprus	848,319	6	8.34301268	8
Estonia	1,315,944	7	10.1547553	10
Latvia	1,968,957	8	12.1618042	12
Slovenia	2,064,188	8	12.4216568	12
Lithuania	2,888,558	11	14.4376944	14
Croatia	4,190,669	12	17.0541533	17
Ireland	4,664,156	13	17.8911687	18
Slovakia	5,407,910	14	19.1161103	19
Finland	5,465,408	14	19.2068166	19
Denmark	5,700,917	14	19.5729496	20
Bulgaria	7,153,784	17	21.6663191	22
Austria	8,711,500	19	23.6635581	24
Hungary	9,830,485	21	24.9787507	25
Sweden	9,998,000	21	25.1683791	25
Portugal	10,341,330	21	25.5516204	25
The Czech Republic	10,445,783	21	25.6668173	26
Greece	10,793,526	21	26.0458098	26
Belgium	11,289,853	21	26.5752358	27
The Netherlands	17,235,349	29	32.1154864	32
Romania	19,759,968	33	34.1418052	34
Poland	37,967,209	52	45.7334937	46
Spain	46,438,422	59	50.0477943	50
Italy	61,302,519	76	56.6715708	57
France	66,661,621	79	58.8378237	59
Germany	82,064,489	96	64.575114	65

## Data Availability

Publicly available datasets were analyzed in this study. This data can be found here: https://ec.europa.eu/eurostat/web/population-demography/demography-population-stock-balance/database and here: https://www.europarl.europa.eu/news/en/press-room/20200130IPR71407/redistribution-of-seats-in-the-european-parliament-after-brexit.
